# *N*,*N*′-Bis(4-nitro­phen­yl)biphenyl-2,2′-dicarboxamide

**DOI:** 10.1107/S1600536810005283

**Published:** 2010-02-17

**Authors:** Gui-Yu Wang, Da-Bin Qin, Li-Hui Guo, Jie-Wei Luo

**Affiliations:** a School of Chemistry and Chemical Engineering, China West Normal University, Nanchong 637002, People’s Republic of China

## Abstract

In the title compound, C_26_H_18_N_4_O_6_, the amide units are approximately coplanar with the benzene ring bonded to the N atom [dihedral angles of 10.59 (10) and 24.00 (12)°], but twisted significantly out of the plane of the benzene ring bonded to the carbonyl C atom [dihedral angles of 57.82 (9) and 58.05 (9)°]. The dihedral angle between the two rings of the biphenyl unit is 77.66 (4)°. Intra­molecular N—H⋯O hydrogen bonds and weak C—H⋯O inter­actions occur. The crystal structure is stabilized by inter­molecular N—H⋯O hydrogen bonds and C—H⋯O contacts.

## Related literature

For the synthesis, see: Gao *et al.* (2002 ▶); Redlich & Hossain (2004 ▶). For related structures, see: Wang *et al.* (2004 ▶); Huang & Yang (2008 ▶).
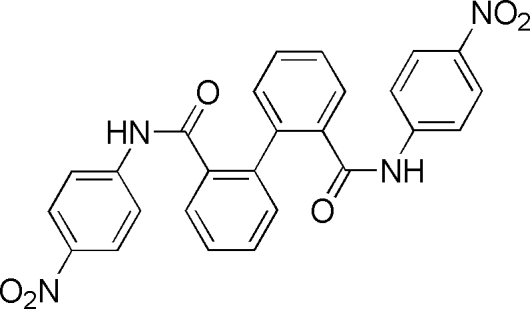


## Experimental

### 

#### Crystal data

C_26_H_18_N_4_O_6_
*M* *_r_* = 482.44
Monoclinic, 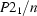

*a* = 12.568 (3) Å
*b* = 12.003 (3) Å
*c* = 14.858 (4) Å
β = 95.324 (2)°
*V* = 2231.8 (11) Å^3^
*Z* = 4
Mo *K*α radiation
μ = 0.11 mm^−1^
*T* = 113 K
0.22 × 0.20 × 0.18 mm

#### Data collection

Rigaku Saturn diffractometer
Absorption correction: multi-scan (*CrystalClear*; Rigaku/MSC, 2004 ▶) *T*_min_ = 0.977, *T*_max_ = 0.981
11020 measured reflections
4108 independent reflections
3412 reflections with *I* > 2σ(*I*)
*R*_int_ = 0.034

#### Refinement

*R*[*F* ^2^ > 2σ(*F* ^2^)] = 0.038
*wR*(*F* ^2^) = 0.098
*S* = 1.02
4108 reflections
334 parameters
2 restraints
H atoms treated by a mixture of independent and constrained refinement
Δρ_max_ = 0.20 e Å^−3^
Δρ_min_ = −0.27 e Å^−3^

### 

Data collection: *CrystalClear* (Rigaku/MSC, 2004 ▶); cell refinement: *CrystalClear*; data reduction: *CrystalClear*; program(s) used to solve structure: *SHELXS97* (Sheldrick, 2008 ▶); program(s) used to refine structure: *SHELXL97* (Sheldrick, 2008 ▶); molecular graphics: *XP* (Sheldrick, 2008 ▶); software used to prepare material for publication: *CrystalStructure* (Rigaku/MSC, 2004 ▶).

## Supplementary Material

Crystal structure: contains datablocks shelxl, I. DOI: 10.1107/S1600536810005283/bt5169sup1.cif

Structure factors: contains datablocks I. DOI: 10.1107/S1600536810005283/bt5169Isup2.hkl

Additional supplementary materials:  crystallographic information; 3D view; checkCIF report

## Figures and Tables

**Table 1:** Hydrogen-bond geometry (Å, °)

*D*—H⋯*A*	*D*—H	H⋯*A*	*D*⋯*A*	*D*—H⋯*A*
N2—H2*A*⋯O4^i^	0.90 (1)	2.01 (1)	2.8763 (17)	160 (1)
N3—H3*A*⋯O3	0.90 (1)	1.99 (1)	2.8878 (17)	178 (1)
C5—H5⋯O3	0.95	2.34	2.9137 (18)	119
C6—H6⋯O1^ii^	0.95	2.59	3.2340 (19)	125
C22—H22⋯O4	0.95	2.22	2.8353 (19)	121

Symmetry codes: (i) 
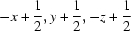
; (ii) 
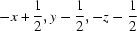
.

**Fig. 1.**
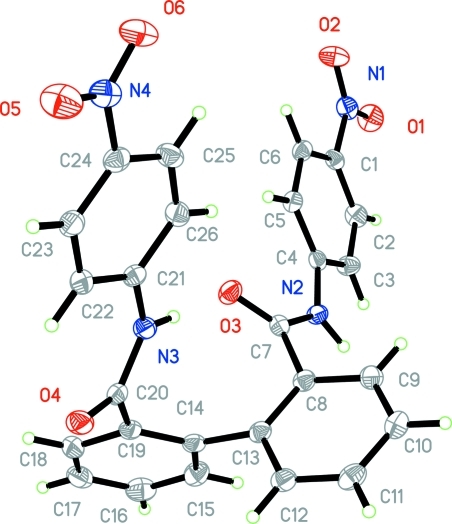
The molecular structure of the title compound, showing 50% probability displacement ellipsoids and the atomic numbering.

## supplementary crystallographic information

### Comment

In the title compound  the  amide units are fairly coplanar with the phenyl ring bonded to the N atom [10.59 (10)° and 24.00 (12)°], but significantly twisted out of the plane of the phenyl ring bonded to the carbonyl C atom [57.82 (9)° and 58.05 (9)°]. The dihedral angle between the two rings of the biphenyl unit is 77.66 (4)°. The crystal structure is stabilized by intramolecular and intermolecular N—H···O hydrogen bonds. In addition, there are short intramolecular and intermolecular C—H···O contacts (Table 1).

### Experimental

The title compound was prepared according to the reported procedure of Gao *et al.* (2002) and Redlich & Hossain (2004). Colorless single crystals suitable for X-ray diffraction were obtained by recrystallization from acetonitrile.

### Refinement

H atoms bonded to C were placed in calculated positions with C—H = 0.95 Å and refined as riding model with *U*_iso_(H) = 1.2*U*_eq_(C). H atoms bonded to N were refined isotropically.

### Crystal data

**Table:**

C_26_H_18_N_4_O_6_	*F*(000) = 1000
*M**_r_* = 482.44	*D*_x_ = 1.436 Mg m^−^^3^
Monoclinic, *P*2_1_/*n*	Mo *K*α radiation, λ = 0.71070 Å
*a* = 12.568 (3) Å	Cell parameters from 5737 reflections
*b* = 12.003 (3) Å	θ = 1.7–27.9°
*c* = 14.858 (4) Å	µ = 0.11 mm^−^^1^
β = 95.324 (2)°	*T* = 113 K
*V* = 2231.8 (11) Å^3^	Prism, colorless
*Z* = 4	0.22 × 0.20 × 0.18 mm

### Data collection

**Table:**

Rigaku Saturn diffractometer	4108 independent reflections
Radiation source: Rotating anode	3412 reflections with *I* > 2σ(*I*)
confocal	*R*_int_ = 0.034
Detector resolution: 7.31 pixels mm^-1^	θ_max_ = 25.5°, θ_min_ = 2.0°
ω scans	*h* = −15→11
Absorption correction: multi-scan (*CrystalClear*; Rigaku/MSC, 2004)	*k* = −13→14
*T*_min_ = 0.977, *T*_max_ = 0.981	*l* = −12→17
11020 measured reflections	

### Refinement

**Table:**

Refinement on *F*^2^	Primary atom site location: structure-invariant direct methods
Least-squares matrix: full	Secondary atom site location: difference Fourier map
*R*[*F*^2^ > 2σ(*F*^2^)] = 0.038	Hydrogen site location: inferred from neighbouring sites
*wR*(*F*^2^) = 0.098	H atoms treated by a mixture of independent and constrained refinement
*S* = 1.02	*w* = 1/[σ^2^(*F*_o_^2^) + (0.0639*P*)^2^] where *P* = (*F*_o_^2^ + 2*F*_c_^2^)/3
4108 reflections	(Δ/σ)_max_ < 0.001
334 parameters	Δρ_max_ = 0.20 e Å^−^^3^
2 restraints	Δρ_min_ = −0.26 e Å^−^^3^

### Special details

**Table:**

Geometry. All e.s.d.'s (except the e.s.d. in the dihedral angle between two l.s. planes) are estimated using the full covariance matrix. The cell e.s.d.'s are taken into account individually in the estimation of e.s.d.'s in distances, angles and torsion angles; correlations between e.s.d.'s in cell parameters are only used when they are defined by crystal symmetry. An approximate (isotropic) treatment of cell e.s.d.'s is used for estimating e.s.d.'s involving l.s. planes.
Refinement. Refinement of *F*^2^ against ALL reflections. The weighted *R*-factor *wR* and goodness of fit *S* are based on *F*^2^, conventional *R*-factors *R* are based on *F*, with *F* set to zero for negative *F*^2^. The threshold expression of *F*^2^ > σ(*F*^2^) is used only for calculating *R*-factors(gt) *etc*. and is not relevant to the choice of reflections for refinement. *R*-factors based on *F*^2^ are statistically about twice as large as those based on *F*, and *R*- factors based on ALL data will be even larger.

### Fractional atomic coordinates and isotropic or equivalent isotropic displacement parameters (Å^2^)

**Table:**

	*x*	*y*	*z*	*U*_iso_*/*U*_eq_	
N1	0.22874 (9)	0.61297 (10)	−0.24831 (8)	0.0245 (3)	
N2	0.17963 (9)	0.37120 (10)	0.06868 (8)	0.0191 (3)	
N3	0.39547 (9)	0.03271 (10)	0.15619 (8)	0.0209 (3)	
N4	0.45085 (10)	−0.31187 (10)	−0.09135 (8)	0.0268 (3)	
O1	0.19449 (8)	0.70898 (9)	−0.25307 (8)	0.0328 (3)	
O2	0.26451 (9)	0.56407 (9)	−0.31202 (7)	0.0331 (3)	
O3	0.30986 (7)	0.23718 (8)	0.07673 (6)	0.0258 (2)	
O4	0.44656 (8)	−0.04883 (8)	0.29288 (7)	0.0261 (2)	
O5	0.48585 (9)	−0.40159 (9)	−0.06211 (8)	0.0390 (3)	
O6	0.42693 (9)	−0.29307 (9)	−0.17211 (7)	0.0350 (3)	
C1	0.22503 (10)	0.55285 (11)	−0.16268 (9)	0.0197 (3)	
C2	0.17265 (11)	0.60088 (12)	−0.09468 (10)	0.0242 (3)	
H2	0.1444	0.6741	−0.1013	0.029*	
C3	0.16220 (11)	0.54058 (12)	−0.01700 (10)	0.0228 (3)	
H3	0.1261	0.5724	0.0302	0.027*	
C4	0.20437 (10)	0.43306 (11)	−0.00715 (9)	0.0176 (3)	
C5	0.26065 (10)	0.38784 (11)	−0.07520 (9)	0.0187 (3)	
H5	0.2923	0.3162	−0.0677	0.022*	
C6	0.27002 (10)	0.44780 (11)	−0.15348 (10)	0.0202 (3)	
H6	0.3071	0.4171	−0.2006	0.024*	
C7	0.22510 (10)	0.27388 (11)	0.10011 (9)	0.0192 (3)	
C8	0.16061 (11)	0.20871 (11)	0.16179 (9)	0.0196 (3)	
C9	0.05533 (11)	0.18147 (12)	0.13161 (10)	0.0228 (3)	
H9	0.0232	0.2130	0.0771	0.027*	
C10	−0.00279 (11)	0.10879 (12)	0.18054 (10)	0.0250 (3)	
H10	−0.0746	0.0910	0.1598	0.030*	
C11	0.04420 (11)	0.06229 (12)	0.25960 (10)	0.0257 (3)	
H11	0.0055	0.0105	0.2923	0.031*	
C12	0.14757 (11)	0.09124 (12)	0.29113 (10)	0.0228 (3)	
H12	0.1785	0.0602	0.3463	0.027*	
C13	0.20747 (11)	0.16517 (11)	0.24363 (9)	0.0198 (3)	
C14	0.31259 (10)	0.20675 (12)	0.28613 (9)	0.0199 (3)	
C15	0.31442 (12)	0.31004 (12)	0.32959 (10)	0.0270 (3)	
H15	0.2511	0.3536	0.3266	0.032*	
C16	0.40666 (13)	0.35024 (14)	0.37692 (10)	0.0308 (4)	
H16	0.4061	0.4206	0.4062	0.037*	
C17	0.49948 (12)	0.28782 (13)	0.38149 (10)	0.0287 (4)	
H17	0.5631	0.3153	0.4134	0.034*	
C18	0.49917 (11)	0.18540 (13)	0.33941 (9)	0.0255 (3)	
H18	0.5627	0.1422	0.3430	0.031*	
C19	0.40649 (11)	0.14455 (11)	0.29155 (9)	0.0199 (3)	
C20	0.41647 (10)	0.03335 (12)	0.24765 (9)	0.0203 (3)	
C21	0.40998 (10)	−0.05654 (11)	0.09698 (9)	0.0195 (3)	
C22	0.46411 (10)	−0.15411 (12)	0.12407 (10)	0.0226 (3)	
H22	0.4922	−0.1633	0.1852	0.027*	
C23	0.47664 (11)	−0.23714 (12)	0.06188 (10)	0.0235 (3)	
H23	0.5132	−0.3039	0.0799	0.028*	
C24	0.43563 (11)	−0.22264 (12)	−0.02687 (9)	0.0224 (3)	
C25	0.38144 (11)	−0.12659 (12)	−0.05546 (10)	0.0247 (3)	
H25	0.3538	−0.1181	−0.1168	0.030*	
C26	0.36826 (11)	−0.04339 (12)	0.00675 (9)	0.0226 (3)	
H26	0.3309	0.0228	−0.0116	0.027*	
H2A	0.1284 (10)	0.3995 (13)	0.1007 (10)	0.030 (4)*	
H3A	0.3673 (12)	0.0956 (10)	0.1315 (10)	0.032 (4)*	

### Atomic displacement parameters (Å^2^)

**Table:**

	*U*^11^	*U*^22^	*U*^33^	*U*^12^	*U*^13^	*U*^23^
N1	0.0246 (6)	0.0244 (7)	0.0242 (7)	−0.0037 (5)	0.0001 (5)	0.0051 (5)
N2	0.0198 (6)	0.0199 (6)	0.0181 (6)	0.0026 (5)	0.0044 (5)	0.0019 (5)
N3	0.0223 (6)	0.0222 (6)	0.0185 (6)	0.0058 (5)	0.0036 (5)	0.0046 (5)
N4	0.0285 (7)	0.0276 (7)	0.0246 (7)	−0.0078 (6)	0.0045 (5)	−0.0019 (6)
O1	0.0371 (6)	0.0241 (6)	0.0371 (6)	0.0037 (5)	0.0034 (5)	0.0123 (5)
O2	0.0468 (7)	0.0330 (6)	0.0202 (6)	−0.0023 (5)	0.0059 (5)	0.0015 (5)
O3	0.0225 (5)	0.0294 (6)	0.0265 (6)	0.0061 (4)	0.0077 (4)	0.0091 (4)
O4	0.0310 (6)	0.0267 (6)	0.0211 (5)	0.0076 (5)	0.0053 (4)	0.0071 (4)
O5	0.0576 (8)	0.0245 (6)	0.0347 (7)	0.0028 (5)	0.0027 (5)	−0.0042 (5)
O6	0.0451 (7)	0.0392 (7)	0.0208 (6)	−0.0076 (5)	0.0039 (5)	−0.0028 (5)
C1	0.0194 (7)	0.0206 (7)	0.0184 (7)	−0.0036 (6)	−0.0012 (5)	0.0036 (6)
C2	0.0277 (8)	0.0183 (7)	0.0261 (8)	0.0037 (6)	0.0001 (6)	0.0027 (6)
C3	0.0274 (8)	0.0204 (7)	0.0211 (7)	0.0031 (6)	0.0046 (6)	−0.0010 (6)
C4	0.0156 (7)	0.0195 (7)	0.0171 (7)	−0.0023 (5)	−0.0013 (5)	0.0012 (6)
C5	0.0177 (7)	0.0168 (7)	0.0216 (7)	0.0002 (6)	0.0020 (5)	0.0013 (6)
C6	0.0180 (7)	0.0210 (7)	0.0220 (7)	−0.0021 (6)	0.0038 (5)	−0.0006 (6)
C7	0.0178 (7)	0.0217 (7)	0.0179 (7)	0.0000 (6)	−0.0002 (5)	0.0007 (6)
C8	0.0203 (7)	0.0184 (7)	0.0205 (7)	0.0034 (6)	0.0044 (6)	0.0018 (6)
C9	0.0228 (8)	0.0233 (8)	0.0223 (7)	0.0030 (6)	0.0024 (6)	0.0011 (6)
C10	0.0206 (7)	0.0240 (8)	0.0309 (8)	−0.0008 (6)	0.0046 (6)	−0.0001 (6)
C11	0.0266 (8)	0.0208 (7)	0.0313 (8)	−0.0004 (6)	0.0113 (6)	0.0035 (6)
C12	0.0251 (8)	0.0216 (7)	0.0225 (7)	0.0045 (6)	0.0060 (6)	0.0042 (6)
C13	0.0204 (7)	0.0192 (7)	0.0203 (7)	0.0049 (6)	0.0055 (5)	0.0011 (6)
C14	0.0221 (8)	0.0224 (7)	0.0158 (7)	0.0005 (6)	0.0048 (5)	0.0049 (6)
C15	0.0274 (8)	0.0248 (8)	0.0298 (8)	0.0026 (6)	0.0071 (6)	0.0000 (7)
C16	0.0397 (9)	0.0268 (8)	0.0268 (8)	−0.0067 (7)	0.0070 (7)	−0.0044 (7)
C17	0.0295 (8)	0.0364 (9)	0.0195 (8)	−0.0081 (7)	−0.0003 (6)	0.0011 (7)
C18	0.0214 (8)	0.0341 (8)	0.0206 (8)	0.0010 (6)	0.0009 (6)	0.0060 (6)
C19	0.0215 (7)	0.0224 (7)	0.0164 (7)	0.0006 (6)	0.0051 (5)	0.0054 (6)
C20	0.0155 (7)	0.0262 (8)	0.0196 (7)	0.0018 (6)	0.0043 (5)	0.0042 (6)
C21	0.0170 (7)	0.0231 (7)	0.0192 (7)	−0.0016 (6)	0.0052 (5)	0.0024 (6)
C22	0.0207 (7)	0.0264 (8)	0.0207 (7)	0.0043 (6)	0.0019 (6)	0.0034 (6)
C23	0.0216 (7)	0.0257 (8)	0.0239 (8)	0.0033 (6)	0.0048 (6)	0.0033 (6)
C24	0.0209 (7)	0.0240 (7)	0.0229 (8)	−0.0043 (6)	0.0062 (6)	−0.0008 (6)
C25	0.0258 (8)	0.0300 (8)	0.0182 (7)	−0.0046 (7)	0.0009 (6)	0.0041 (6)
C26	0.0229 (7)	0.0232 (7)	0.0218 (8)	0.0013 (6)	0.0019 (6)	0.0062 (6)

### Geometric parameters (Å, °)

**Table:**

N1—O1	1.2299 (16)	C10—C11	1.383 (2)
N1—O2	1.2337 (16)	C10—H10	0.9500
N1—C1	1.4672 (18)	C11—C12	1.384 (2)
N2—C7	1.3636 (18)	C11—H11	0.9500
N2—C4	1.4080 (17)	C12—C13	1.3966 (19)
N2—H2A	0.902 (9)	C12—H12	0.9500
N3—C20	1.3601 (18)	C13—C14	1.4962 (19)
N3—C21	1.4088 (18)	C14—C19	1.3925 (19)
N3—H3A	0.898 (9)	C14—C15	1.397 (2)
N4—O5	1.2273 (17)	C15—C16	1.386 (2)
N4—O6	1.2306 (16)	C15—H15	0.9500
N4—C24	1.4613 (19)	C16—C17	1.383 (2)
O3—C7	1.2322 (16)	C16—H16	0.9500
O4—C20	1.2330 (17)	C17—C18	1.379 (2)
C1—C2	1.3821 (19)	C17—H17	0.9500
C1—C6	1.3835 (19)	C18—C19	1.396 (2)
C2—C3	1.379 (2)	C18—H18	0.9500
C2—H2	0.9500	C19—C20	1.496 (2)
C3—C4	1.3977 (19)	C21—C22	1.3948 (19)
C3—H3	0.9500	C21—C26	1.402 (2)
C4—C5	1.3964 (18)	C22—C23	1.378 (2)
C5—C6	1.3820 (19)	C22—H22	0.9500
C5—H5	0.9500	C23—C24	1.382 (2)
C6—H6	0.9500	C23—H23	0.9500
C7—C8	1.4988 (18)	C24—C25	1.386 (2)
C8—C9	1.396 (2)	C25—C26	1.381 (2)
C8—C13	1.4021 (19)	C25—H25	0.9500
C9—C10	1.3862 (19)	C26—H26	0.9500
C9—H9	0.9500		
			
O1—N1—O2	123.52 (12)	C11—C12—C13	121.44 (14)
O1—N1—C1	118.14 (12)	C11—C12—H12	119.3
O2—N1—C1	118.33 (12)	C13—C12—H12	119.3
C7—N2—C4	127.52 (11)	C12—C13—C8	118.19 (13)
C7—N2—H2A	116.1 (10)	C12—C13—C14	119.67 (12)
C4—N2—H2A	116.4 (10)	C8—C13—C14	121.67 (12)
C20—N3—C21	127.15 (12)	C19—C14—C15	118.14 (13)
C20—N3—H3A	115.9 (11)	C19—C14—C13	123.78 (13)
C21—N3—H3A	116.9 (11)	C15—C14—C13	117.85 (12)
O5—N4—O6	123.58 (13)	C16—C15—C14	121.38 (14)
O5—N4—C24	118.39 (12)	C16—C15—H15	119.3
O6—N4—C24	118.03 (13)	C14—C15—H15	119.3
C2—C1—C6	121.73 (13)	C17—C16—C15	119.90 (15)
C2—C1—N1	118.98 (12)	C17—C16—H16	120.0
C6—C1—N1	119.24 (12)	C15—C16—H16	120.0
C3—C2—C1	118.81 (13)	C18—C17—C16	119.60 (14)
C3—C2—H2	120.6	C18—C17—H17	120.2
C1—C2—H2	120.6	C16—C17—H17	120.2
C2—C3—C4	120.51 (13)	C17—C18—C19	120.74 (14)
C2—C3—H3	119.7	C17—C18—H18	119.6
C4—C3—H3	119.7	C19—C18—H18	119.6
C5—C4—C3	119.71 (12)	C14—C19—C18	120.24 (13)
C5—C4—N2	122.60 (12)	C14—C19—C20	123.93 (13)
C3—C4—N2	117.45 (12)	C18—C19—C20	115.83 (12)
C6—C5—C4	119.70 (13)	O4—C20—N3	124.15 (13)
C6—C5—H5	120.1	O4—C20—C19	120.66 (12)
C4—C5—H5	120.1	N3—C20—C19	115.11 (12)
C5—C6—C1	119.46 (13)	C22—C21—C26	119.97 (13)
C5—C6—H6	120.3	C22—C21—N3	123.10 (13)
C1—C6—H6	120.3	C26—C21—N3	116.93 (12)
O3—C7—N2	123.74 (12)	C23—C22—C21	119.78 (13)
O3—C7—C8	121.10 (12)	C23—C22—H22	120.1
N2—C7—C8	115.04 (11)	C21—C22—H22	120.1
C9—C8—C13	120.04 (12)	C22—C23—C24	119.55 (13)
C9—C8—C7	118.43 (12)	C22—C23—H23	120.2
C13—C8—C7	121.16 (12)	C24—C23—H23	120.2
C10—C9—C8	120.59 (14)	C23—C24—C25	121.78 (14)
C10—C9—H9	119.7	C23—C24—N4	118.26 (13)
C8—C9—H9	119.7	C25—C24—N4	119.95 (13)
C11—C10—C9	119.69 (13)	C26—C25—C24	118.85 (13)
C11—C10—H10	120.2	C26—C25—H25	120.6
C9—C10—H10	120.2	C24—C25—H25	120.6
C10—C11—C12	119.98 (13)	C25—C26—C21	120.06 (13)
C10—C11—H11	120.0	C25—C26—H26	120.0
C12—C11—H11	120.0	C21—C26—H26	120.0
			
O1—N1—C1—C2	7.31 (19)	C12—C13—C14—C15	−96.39 (16)
O2—N1—C1—C2	−171.63 (12)	C8—C13—C14—C15	75.56 (17)
O1—N1—C1—C6	−175.39 (12)	C19—C14—C15—C16	0.0 (2)
O2—N1—C1—C6	5.67 (18)	C13—C14—C15—C16	174.64 (13)
C6—C1—C2—C3	−2.2 (2)	C14—C15—C16—C17	0.2 (2)
N1—C1—C2—C3	175.07 (12)	C15—C16—C17—C18	−0.5 (2)
C1—C2—C3—C4	0.3 (2)	C16—C17—C18—C19	0.6 (2)
C2—C3—C4—C5	2.2 (2)	C15—C14—C19—C18	0.05 (19)
C2—C3—C4—N2	−172.40 (13)	C13—C14—C19—C18	−174.22 (12)
C7—N2—C4—C5	17.4 (2)	C15—C14—C19—C20	−179.36 (12)
C7—N2—C4—C3	−168.16 (13)	C13—C14—C19—C20	6.4 (2)
C3—C4—C5—C6	−2.92 (19)	C17—C18—C19—C14	−0.4 (2)
N2—C4—C5—C6	171.38 (12)	C17—C18—C19—C20	179.09 (12)
C4—C5—C6—C1	1.14 (19)	C21—N3—C20—O4	−4.3 (2)
C2—C1—C6—C5	1.4 (2)	C21—N3—C20—C19	172.44 (12)
N1—C1—C6—C5	−175.80 (12)	C14—C19—C20—O4	−123.24 (15)
C4—N2—C7—O3	15.2 (2)	C18—C19—C20—O4	57.33 (17)
C4—N2—C7—C8	−160.85 (12)	C14—C19—C20—N3	59.93 (17)
O3—C7—C8—C9	−121.93 (15)	C18—C19—C20—N3	−119.51 (13)
N2—C7—C8—C9	54.26 (17)	C20—N3—C21—C22	−9.9 (2)
O3—C7—C8—C13	51.05 (19)	C20—N3—C21—C26	170.91 (12)
N2—C7—C8—C13	−132.75 (13)	C26—C21—C22—C23	0.2 (2)
C13—C8—C9—C10	−1.9 (2)	N3—C21—C22—C23	−178.94 (12)
C7—C8—C9—C10	171.13 (13)	C21—C22—C23—C24	0.2 (2)
C8—C9—C10—C11	−0.4 (2)	C22—C23—C24—C25	−0.3 (2)
C9—C10—C11—C12	2.2 (2)	C22—C23—C24—N4	179.58 (12)
C10—C11—C12—C13	−1.5 (2)	O5—N4—C24—C23	9.98 (19)
C11—C12—C13—C8	−0.9 (2)	O6—N4—C24—C23	−170.63 (12)
C11—C12—C13—C14	171.37 (12)	O5—N4—C24—C25	−170.15 (13)
C9—C8—C13—C12	2.56 (19)	O6—N4—C24—C25	9.23 (19)
C7—C8—C13—C12	−170.31 (12)	C23—C24—C25—C26	0.0 (2)
C9—C8—C13—C14	−169.51 (12)	N4—C24—C25—C26	−179.89 (12)
C7—C8—C13—C14	17.62 (19)	C24—C25—C26—C21	0.4 (2)
C12—C13—C14—C19	77.90 (17)	C22—C21—C26—C25	−0.5 (2)
C8—C13—C14—C19	−110.15 (15)	N3—C21—C26—C25	178.68 (12)

### Hydrogen-bond geometry (Å, °)

**Table:**

*D*—H···*A*	*D*—H	H···*A*	*D*···*A*	*D*—H···*A*
N2—H2A···O4^i^	0.90 (1)	2.01 (1)	2.8763 (17)	160 (1)
N3—H3A···O3	0.90 (1)	1.99 (1)	2.8878 (17)	178 (1)
C5—H5···O3	0.95	2.34	2.9137 (18)	119
C6—H6···O1^ii^	0.95	2.59	3.2340 (19)	125
C22—H22···O4	0.95	2.22	2.8353 (19)	121

Symmetry codes: (i) −*x*+1/2, *y*+1/2, −*z*+1/2; (ii) −*x*+1/2, *y*−1/2, −*z*−1/2.
